# Implementation of the Configuration Structure of an Integrated Computational Core of a Pulsed NQR Sensor Based on FPGA

**DOI:** 10.3390/s21186029

**Published:** 2021-09-09

**Authors:** Andriy Samila, Oleksandra Hotra, Jacek Majewski

**Affiliations:** 1 Department of Radio Engineering and Information Security, Yuriy Fedkovych Chernivtsi National University, Kotsyubynsky 2, 58000 Chernivtsi, Ukraine; a.samila@chnu.edu.ua; 2 Department of Electronics and Information Technology, Lublin University of Technology, Nadbystrzycka 38D, 20-618 Lublin, Poland; 3 Department of Automation and Metrology, Lublin University of Technology, Nadbystrzycka 38D, 20-618 Lublin, Poland; j.majewski@pollub.pl

**Keywords:** digital filter, digital down conversion, direct digital synthesis, FPGA, nuclear quadrupole resonance, phase accumulator, sensor, software-defined radio

## Abstract

This paper presents a method for implementing the configuration structure of an integrated computational core of a pulsed nuclear quadrupole resonance (NQR) sensor based on a field-programmable gate array (FPGA), which comprises the following modules: a three-channel direct digital synthesizer (DDS), a pulse sequence shaper and a software-defined radio. Experimental studies carried out using the in-circuit analyzer SignalTap Logic Analyzer have confirmed the reliability of the correct and stable operation of the functional modules of the configuration structure at all stages of signal transformations, starting from the formation of the envelope of the excitation pulses and ending with the obtainment of low-frequency quadrature signals at the outlet of the compensating filters. The time and frequency dependences of the amplitude of the output signals generated using the DDS based on a 48 bit phase accumulator are investigated. This development can be used when creating pulsed coherent NQR sensors in the frequency range of 1 MHz–50 MHz.

## 1. Introduction

The methods of pulsed Fourier and relaxation spectroscopy of NQR are gaining relevance for the remote detection of resonant signals of the ^14^N isotope in solids (drugs and explosives in non-metallic containers) [1]. The results obtained with NQR are also unique in the study of complex multiplet spectra that are characteristic of layered semiconductors due to the polytypic modifications of their crystal structure [2]. Basic and applied research in the field of NQR spectroscopy is carried out in the following topical areas: the development of experimental methods and equipment for NQR observation; the development of hardware and software for the digital processing of spin induction signals in real time; studies of the intramolecular structure of substances and their physicochemical properties; the development of NQR detectors of explosives and drugs; NQR tomography, and the like [1,2,3,4,5,6]. This confirms the relevance of the topic of this paper.

A great contribution to the development of the theoretical foundations and the scientific and technical basis of NQR spectroscopy was made by the scientists R.T. Pound, F.N.H. Robinson, Michael S. Adler, Tara P. Das and V.S. Grechishkin. Research centers in the USA, Germany, Japan, China, and other countries are deeply involved in experimental developments. Marketing research of the existing NQR spectroscopy methods has shown that serial equipment for NQR analysis is represented by only a few leading corporations—SpinCore, Tecmag, Bruker, Varian, and others. The cost of such measuring systems reaches hundreds of thousands of conventional units, which to some extent complicates their use in the research centers of higher education institutions.

Among the main developments, it is worth noting the work on the scientific and practical developments of pulsed NQR spectroscopy equipment [7,8,9,10,11]. However, due to the relevance of the information, many articles on this topic remain closed. One of a small number of open publications provides a generalized review of promising solutions for detecting explosives based on the ^14^N NQR isotope, from which it follows that free induction decay (FID) signals are so weak that they are difficult to detect against the background of the intrinsic noise of the electronic components of circuits [7]. One of the key techniques employed in NQR to increase the signal-to-noise ratio is the use of special multi-pulse sequences of excitation pulses [12]. This technique not only makes it possible to determine the duration of relaxation processes in the NQR, but also allows for the sensitivity of the experiment to increase. That is why one of the key features of modern pulsed NQR sensors is the use of digital signal processing algorithms implemented on the basis of signal processors or an FPGA [10].

Therefore, promising in this direction is the development of inexpensive portable sensors with integrated computational cores, based on new algorithms for information transformations of the FID signal in the transmit and receive path and the visualization of NQR spectra [13,14,15]. At the same time, considerable attention should be paid to increasing the signal-to-noise ratio value provided by the sensor to the required level for error-free detection of the NQR signal in the investigated substances of small volumes with masses from tenths to several grams, and to the development of highly efficient methods for the formation of special multi-pulse sequences of nanosecond time intervals for the stochastic NQR technique [16,17,18].

In this paper, on one FPGA, we propose the implementation of the configuration structure of the original computational core of the NQR sensor, which will reduce the delay time of the signal transmission between individual functional blocks and will make it easy to change their configuration depending on the experimental conditions. Other features of the NQR sensor computational core are the reduced power consumption, cost, size and the increased flexibility. The development of FPGA projects requires deep knowledge of both the theoretical processes and the experimental methods in the fields of NQR spectroscopy, digital synthesis and digital signal processing, so the authors of this article decided to share their experience as it will be useful to many developers and scientists. 

## 2. Basic Structure of an NQR Sensor

### 2.1. Hardware

In modern NQR observation equipment, special devices, namely pulse sequence shapers, are used to generate exciting radio pulses [19,20]. Such devices can be made in the form of special expansion cards for personal computers, or in the form of separate hardware units that connect to other units of the sensor: a radio frequency (RF) transmitter, a receive path, a synchronization unit and a data acquisition system (DAQ). One of the most important characteristics of pulse sequence shapers is the accuracy of the formation of the duration of excitation radio pulses and the stability of their filling frequency. We already successfully developed an FPGA-based pulse sequence shaper for the NQR portable sensor [19]. In [21], we considered the implementation of a cheap digital pulse coherence spectrometer for the study of NQR in GaSe- and InSe-layered semiconductor crystals. We made some modifications to the structure of the pulsed single-coil coherent NQR observation method, which led to the following improvements:

(1) By implementing the pulsed mode of the transistor bias control, the power consumption of the RF transmitter is reduced by almost 50%, and can now operate in pulsed modes (up to 30 ms) even without active cooling, while maintaining the output power up to 1 kW.
(2) The noise level of the pre-amplification stages of the FID signal is reduced by more than 6 times.
(3) The level of carrier oscillation suppression is increased to 100 dB with non-uniformity ±10 dB in the frequency range of 1 –50 MHz.
(4) The possibility of automatic monitoring of the matching of the output impedance of the RF transmitter and the input impedance of the NQR sensor is provided.

**Figure 1:**
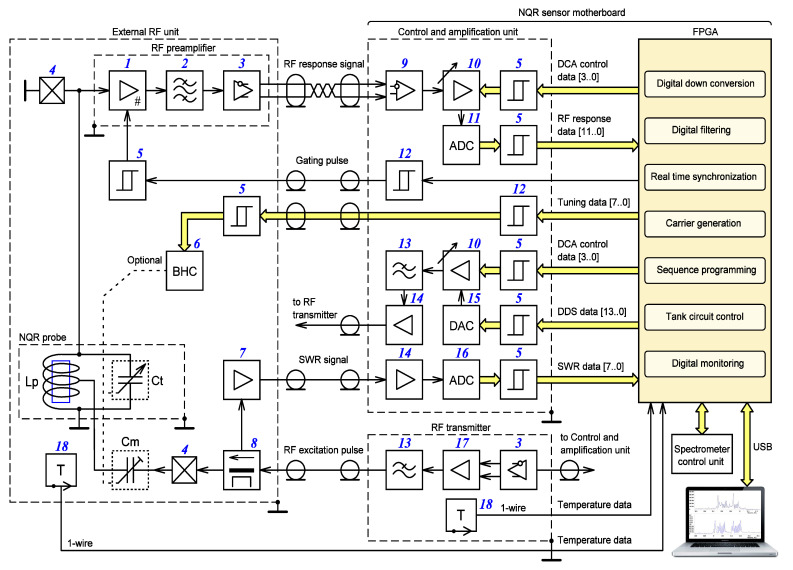
Block diagram of an NQR sensor: (1) gated amplifier; (2) bandpass filter; (3) differential driver; (4) diode limiter; (5) non-inverting buffer; (6) binary–hexadecimal converter; (7) driver amplifier; (8) reflection measurement circuit; (9) differential buffer; (10) digital controlled amplifier; (11) 12 bit analog to digital converter; (12) non-inverting driver; (13) low-pass filter; (14) buffer amplifier; (15) 14 bit digital to analog converter; (16) 8 bit analog to digital converter; (17) radio frequency power amplifier; (18) temperature sensor; (Cm) matching capacitor; (Ct) tuning capacitor; (Lp) probe coil.

Figure 1 shows a block diagram of a new NQR sensor, which reflects the above changes. Information on the electronic components used in the manufacture of the sensor motherboard is given in Section 4.

### 2.2. Basic FPGA Architecture

**Figure 2:**
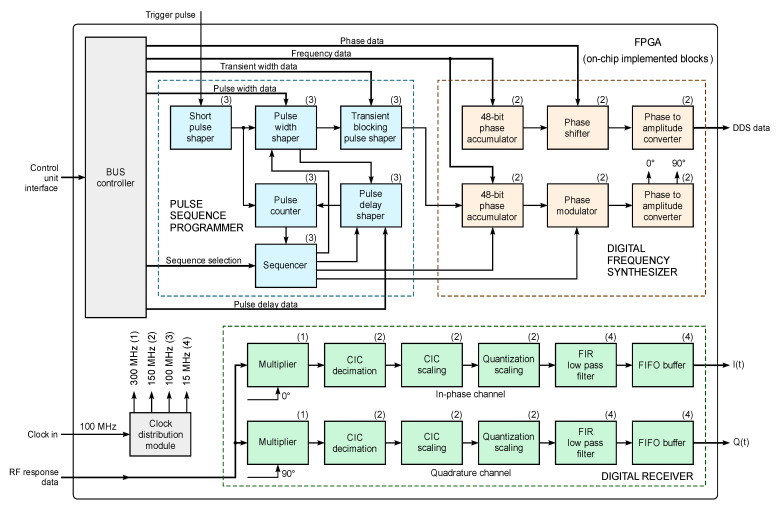
Overall block diagram of FPGA (on-chip implemented blocks).

Consider the block diagram (Figure 2), which shows the configuration of the FPGA architecture for the implementation of algorithms for the digital synthesis of NQR excitation signals and the radio frequency response processing FID. The main units implemented on the chip can be divided by functional purpose into the following categories: programming of pulse sequences, digital frequency synthesis and digital reception.

The pulse sequencer allows the formation of all the time intervals of the enveloping single excitation pulses, or their sequences (pulse duration, pause duration and transient duration are regulated in steps of 100 ns), as well as gating pulses to control the preamplifier and transmitter. The operation of the programmer also takes into account the possibility of setting a small elongation of each generated gating pulse and adjusting its duration to suppress the transient process in the receiving coil of the sensor after the action of the excitation pulse. The digital sequencer, implemented on the basis of non-volatile FPGA memory modules, is used for pre-programming and operative selection of the required sequence of excitation pulses: single RF pulse, Carr–Purcell, Meiboom–Gill, MREV-8, etc.

The DDS generates harmonic periodic signals in the frequency range from 1 MHz to 50 MHz, which serve as carrier oscillations in the formation of radio pulses and their sequences and are also necessary for the operation of software-defined radio (SDR) mixers. The digital part of the DDS is a numerically controlled oscillator (NCO), which is based on two 48 bit phase accumulators. The values of the NCO output signal samples are recorded in the recoding tables of the phase-amplitude transducers implemented on the basis of a non-volatile FPGA memory. The tabular values of one period of each of the harmonic oscillations, which change in time according to the laws *y* = sin(*x*) and *y* = cos(*x*), consist of 2048 14 bit samples. A coherent method of generating NQR excitation signals is provided by synchronizing the initial phase of the leading edge of the envelope of the radio pulses and their sequences with the initial phase of the carrier oscillation generated by the first DDS channel.

SDR, developed on the principle of a digital down-converter (DDC), performs the function of quadrature amplitude demodulation and filtering of the FID signal. This solution makes it possible to significantly reduce the length of the analog path of the NQR sensor, and thus to reduce the noise of the FID signal and the asymmetry of the parameters of its quadrature components. The obtained data come from the SDR outputs to the external hardware module DAQ through the corresponding FIFO buffers. For synchronous operation of all digital synthesis and processing modules, a clock distribution module is used, which operates from an external generator with a frequency of 100 MHz and provides the clock speeds: 300 MHz—for the SDR mixers; 150 MHz—for the operation of the DDS, decimation filters, analog-to-digital and digital-to-analog converters (ADC and DAC) and 15 MHz—for the operation of the SDR compensating filters.

A detailed description of the methods of configuring these modules is provided in the next section.

## 3. Methods of Configuring Devices Based on the Syntax of Modeling Dynamic Modes of Logical Structures

**Figure 3:**
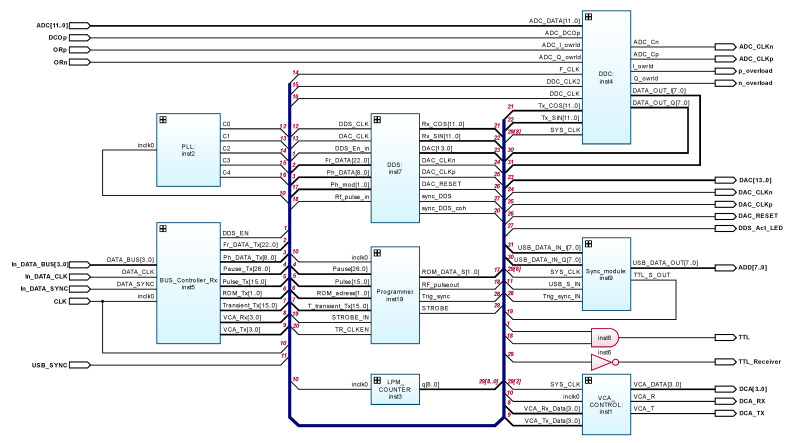
Configuration structure of an integrated computational core of pulsed NQR sensor based on FPGA.

For the implementation, the FPGA of the Cyclone IV family was used, which is characterized by a satisfactory performance at a low cost and a very low power consumption. The following is a description of the main functional modules of the configuration structure, shown in Figure 3:

VCA_Control:inst1—controller of code-controlled amplifiers;

PLL:inst2—clock frequency generation system;

LPM_Counter:inst3—frequency divider;

DDC:inst4—(SDR);

BUS_Controller_Rx:inst5—service controller of the main interface bus, which allows microcontroller control of the computational core;

DDS:inst7—three-channel DDS;

Sync_module2:inst9—synchronization module;

LPM_Constant:inst12, LPM_Constant:inst13—constants;

Programmer:inst19—pulse sequence programmer. 

A characteristic feature of the proposed computational core is the implementation of a pulse shaper based on a three-channel DDS (inst7 in Figure 3), the main channel of which allows operation in a high-speed phase and frequency modulation mode, and the other two channels are used to generate reference signals necessary for the operation of integrated SDR mixers (inst4 in Figure 3). The multi-channel phase-locked loop system (inst2 in Figure 3) performs the functions of a clock distribution module.

Consider in more detail the main modules of the configuration structure, shown in Figure 3.

### 3.1. Pulse Sequence Programmer

**Figure 4:**
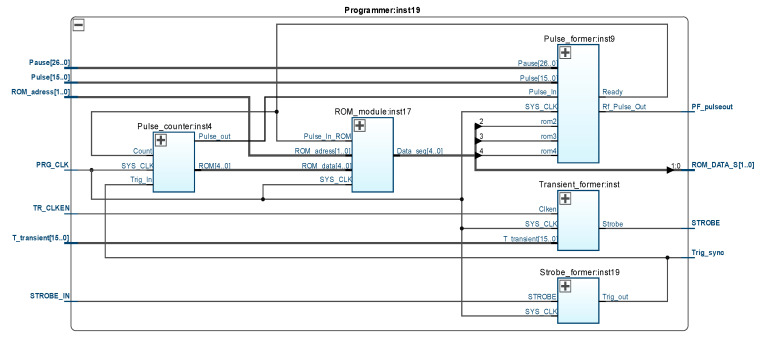
Configuration structure of pulse sequence programmer based on FPGA.

**Figure 5:**
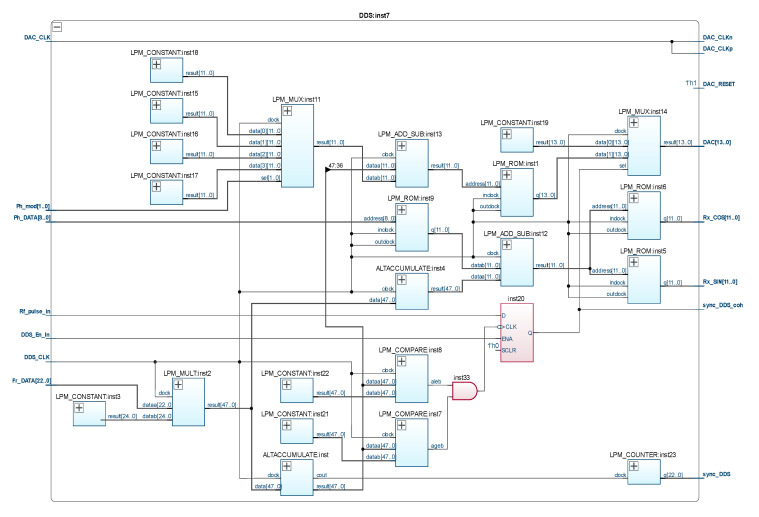
Configuration structure of digital frequency synthesizer based on FPGA.

The configuration structure of the Programmer:inst19 module, which provides the formation of individual video pulses or their sequences, is shown in Figure 4. In fact, the considered module serves to control the first DDS channel (Figure 5), allowing the formation of excitation radio pulses of appropriate durations. Changing the logic level at the “Strobe_In” input starts the process of shaping the strobe pulse by the Strobe_former:inst19 module. This pulse activates the pulse counter in the sequence Pulse_counter:inst4, the source code of which from the output “ROM[4..0]” is fed to the non-volatile memory module ROM_module: inst17, and changing the level at the output “Pulse_out” leads to the activation of the video pulse generator Pulse_former:inst9. The operating principle of the latter is described in detail in [19]. Code words with data on the duration of the 90° pulse and the pause between the first and second pulses in the sequence are sent from the main interface controller (inst5 in Figure 3) to the inputs “Pulse[15..0]” and “Pause[26..0]”, respectively. This ensures the formation of the 90° radio pulse with a duration of 0.1 μs–100 μs and the first pause with a duration of 0.1 μs–500 ms [22]. Data on the duration of other pulses and pauses in the complex specialized sequences for NQR excitation and spin echo study are stored in non-volatile memory (inst17 in Figure 4) and read in accordance with the code at the “ROM_adress[1..0]” input. The total number of pulses in the sequence is not fixed and, due to the ability to quickly change the FPGA configuration, can be set depending on the experimental conditions. The Transient_former:inst module serves to further increase the duration of the excitation pulses due to the need to close the receiver of the sensor during the action of the transient process in its input circuit [23]. Additional gain in duration can be set in the range of 0.1 μs–10 μs.

### 3.2. Frequency Synthesizer

As one of the main requirements in the formation of NQR excitation pulses is the stability of the carrier frequency and the ability to quickly reconfigure the amplitude, frequency and phase, it was decided, with regard to the results of our previous analytical studies, to use the tabular method of direct digital frequency synthesis to generate radio frequency oscillations [24,25].

Consider the configuration structure of the FPGA-based DDS (Figure 5), in particular its first channel, which is used to generate NQR RF excitation pulses. The output quasi-harmonic signal of the first channel of the synthesizer is formed by an external 14 bit digital-to-analog converter connected to the “DAC[13..0]” output. The digital code is read from the read-only memory (ROM) LPM_ROM:inst1, in the cells of which the values of the coefficients of the sinusoidal signal are pre-written, and whose capacity determines the number of steps of the approximated sinusoid. The 48 bit phase accumulator ALTACCUMULATE:inst is a register whose content increases linearly over time with an adjustable increment. In this case, the binary code at the output “result[47..0]” of the phase accumulator is the code of the instantaneous phase of the generated signal, the frequency of which is proportional to the rate of phase change in time, and the phase gain is the code of the output frequency.

The minimum step of adjusting the frequency of the DDS output signal is determined by the expression [26]:

(1) $$\Delta f_{out}=\frac{f_{clk}}{2^{M}},$$

where *f_clk_* is clock frequency and *M* is phase accumulator capacity.

To change the frequency of the DDS output signal, you must set the appropriate phase gain. If *f_clk_ =* 150 MHz and *M* = 48 bit and the phase gain *K* is equal to unity, then Δ*f_out_ =* 5.329 × 10^−7^ Hz.

Thus, by changing *K*, you can set the frequency of the output signal by the expression [26]:

(2) $$f_{out}=\frac{K\times f_{clk}}{2^{M}}.$$

The LPM_Mult:inst2 module performs the function of multiplying the binary code, which is fed to the input “Fr_Data[22..0]” by the constant LPM_Constant:inst3, thus generating the frequency code for the phase accumulators ALTACCUMULATE:inst and ALTACCUMULATE:inst4 of the main (first) and auxiliary (second and third) channels of the synthesizer, respectively.

The other two channels of the configuration structure, shown in Figure 5, operate in the same way as the main channel. They operate at the frequency of the main channel and are used to generate the quadrature signals (outputs “Rx_cos[11..0]” and “Rx_sin[11..0]”) required for the operation of a digital receiver.

The key difference of the proposed solution is the ability to readjust the phase of the signal generated by the first channel with an accuracy of 1 degree and synchronize the initial phase of the generated radio pulses, with the leading edges of the video pulses arriving at the RF_pulse_In input from the output of the pulse train programmer (Figure 5). The latter makes it possible to avoid the time-increasing errors of establishing the duration of the 90° and 180° excitation pulses of NQR, which occur when using classical incoherent techniques of radio pulse formation [19,27]. The modules LPM_Constant:inst15—LPM_Constant:inst18 and LPM_MUX:inst11 are used to provide quadrature phase manipulation. Additional adder LPM_Add_Sub:inst13 provides the ability to set the phase of the signal generated by the first synthesizer channel in accordance with the phase code, which is fed to the “Ph_Data[8..0]” input.

### 3.3. Digital Receiver

**Figure 6:**
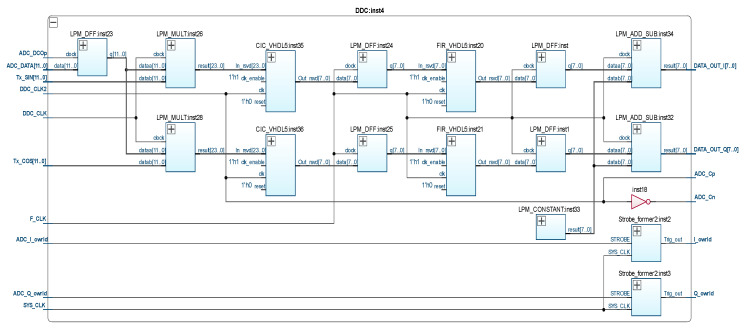
Configuration structure of digital receiver based on FPGA.

To implement the configuration structure of a digital receiver based on an FPGA, its simulation model was developed. In the MATLAB Simulink software, the simulation and calculation of the parameters of its main modules, in particular the characteristics and coefficients of the digital filters, were carried out [28]. The theoretical basis of the simulation model is based on the SDR method, the essence of which is the use of full digitization of the FID signal by high-speed ADCs with subsequent processing of the received data by a digital signal processor [29]. In this case, the basic parameters of the receiver are determined precisely by the software, and not by the hardware configuration and, therefore, they can be easily reconfigured. The configuration structure of a digital receiver operating on the principle of a DDC is shown in Figure 6.

Digital data from the 12 bit DAC output through the “ADC_Data[11..0]” input and the LPM_DFF:inst23 register are fed to the input of the LPM_Mult:inst26 and LPM_Mult:inst28 multipliers. The isolation of the complex envelope of the FID signal ensures the transfer of the spectrum to the region of zero frequencies and leads to the formation of a signal that can be described by the formula [30]:

(3) $$z_{d}(t)=s(t)\exp(-j\omega_{0}t)=A(t)+jB(t),$$

where the complex components [30]:

(4) $$\begin{array}{l} A(t)=\frac{1}{2}I(t)+\frac{1}{2}I(t)\cos(2\omega_{0}t)-\frac{1}{2}Q(t)\sin(2\omega_{0}t),\\ B(t)=\frac{1}{2}Q(t)-\frac{1}{2}Q(t)\cos(2\omega_{0}t)-\frac{1}{2}I(t)\sin(2\omega_{0}t). \end{array}$$

The CIC_VHDL5:inst35 and CIC_VHDL:inst36 modules are five-stage integral-comb filters with infinite pulse response, used to reduce the bit rate to 8 bit and the sampling rate to 15 MHz signals from the multiplier outputs. The latter allows the filtering of the demodulated signal from the spectral components of the higher orders [29]. The uneven amplitude–frequency response of CIC filters necessitates the use of additional compensating filters. The proposed configuration structure of the digital receiver uses non-recursive compensating filters of the 55th order with a final pulse response (modules FIR_VHDL5:inst20 and FIR_VHDL5:inst21 in Figure 6). The data coefficients of FIR filters are calculated in MATLAB FDATool. Earlier, we described the processes of simulation modeling and the synthesis of SDR filters in MATLAB Simulink in more detail [28,29].

## 4. Hardware Implementation and Experiment

**Figure 7:**
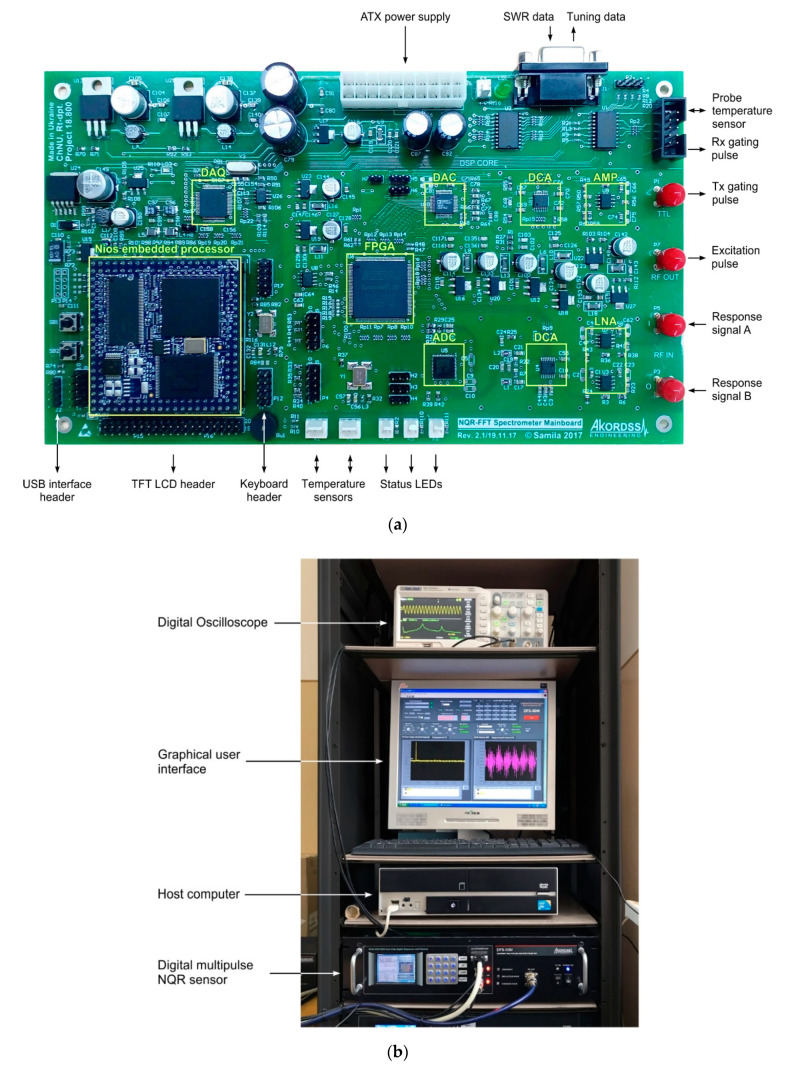
Photo of the experimental setup for the study of the proposed configuration structure of the FPGA: (**a**) view of the NQR sensor motherboard; (**b**) measurement setup for the observation of NQR.

As a hardware base for the implementation of the proposed configuration structure, we used the motherboard of a pulsed NQR sensor (Figure 7a), the operating principle of which is described in detail in [21,31]. The core of the motherboard is an FPGA EP4CE15E22C8, the internal structure of which contains more than 15,000 logic gates. The peculiarity of the applied FPGA is the presence of four functionally independent PLL modules in it. According to official data, the maximum clock frequency for FPGAs of the Cyclone IV C8 series is 402 MHz, which is quite enough for the operation of all the digital modules of the proposed configuration structure. Table 1 summarizes the information for connecting the motherboard hardware to the FPGA I/O ports.

A high-speed 12 bit ADC AD9230BCPZ (conversion rate of up to 210 MSPS) was used to digitize the FID signal, and a high-speed 14 bit DAC AD9772AASTZ (conversion rate of up to 150 MSPS) with built-in interpolation filters was used to output the synthesized signal from the outlet of the first DDS channel. In the previous stages of the analog amplifier path, low-noise (0.95 nV/√Hz) operational amplifiers LT6201 were used. The output resistance of the device is matched to the input impedance of the high-frequency power amplifier using an operational amplifier AD8055ARZ with a bandwidth of 300 MHz. Separate control of the amplitudes of the input and output signals is carried out by amplifiers controlled by the code AD8369ARU, the gain of which is set by software from the FPGA. Two TTL-compatible synchronization channels, developed using the high-speed logic buffers 74VHCT244ADW, provide transmission of control signals to the hardware modules of the transmitter and the receiver of the NQR sensor. The sensor motherboard has connectors for connecting the transmit and receive paths of the sensor, as well as the indicating and control elements. Data exchange with a computer via a USB interface is provided by a hardware USB-FIFO controller FT2232HL.

The setup for the experimental studies of the FPGA configuration structure is shown in Figure 7b.

The configuration loaded in the FPGA can be stored in both static memory (SRAM) and non-volatile storage (EEPROM or Flash). The EP4CE15E22C8 chip belongs to the SRAM configuration FPGAs, so the configuration information is stored by using an external EPCS4 chip with a serial interface. The developed device has two programming modes: Active Serial and via JTAG-interface. The choice of configuration mode is determined by the combination on the MSEL inputs of the FPGA chip.

According to the results of the compilation in the Quartus Prime software of the project of the configuration structure of the digital computing core of the NQR sensor, the following data on the use of FPGA resources were obtained: total logic elements—9.642/15.408 (63%); total pins—61/82 (74%); total memory bits—223.400/516,096 (44%); embedded multiplier 9 bit elements—9/112 (8%) and total PLLs—1/4 (25%).

With minor design changes, you can also use any Intel debug board on a Cyclone FPGA of at least generation IV with a sufficient number of logic elements and I/O ports. In this case, you will also need to provide high-speed ADCs and DACs.

To debug the proposed configuration structure, the means of the in-circuit analyzer SignalTap Logic Analyzer were used [32]. The peculiarity of this tool is the possibility of its implementation together with the developed configuration structure on one FPGA, which, as a result, provides the presence of the shortest possible short circuits and the ability to work in real time.

**Figure 8:**
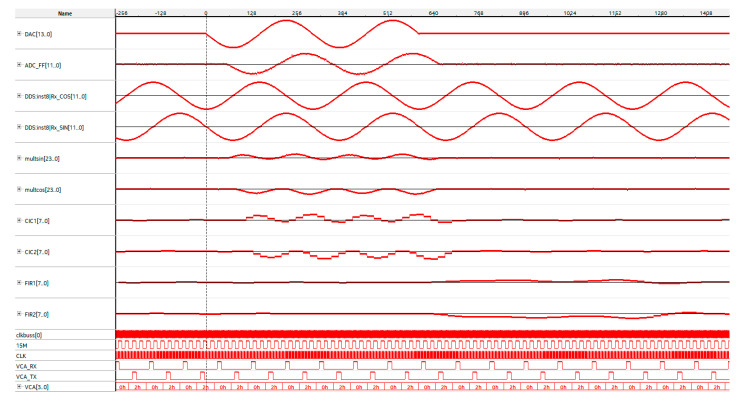
The results of the study of the proposed configuration structure obtained using the in-circuit analyzer SignalTap Logic Analyzer.

The results of investigating the operating modes of the main modules of the configuration structure of an integrated computational core of the pulsed NQR sensor are shown in the form of signalograms in Figure 8.

Consider the most relevant dependences. The DAC[13..0] signalogram visualizes a coherent 90° radio pulse with a frequency of 1 MHz and a duration of 2 μs, synthesized at the outlet of the first DDS channel controlled by the pulse sequence programmer. Quadrature signals at the outlets of the second and third DDS channels are represented by the signalograms DDC:inst18|Rx_cos[11..0] and DDC:inst18|Rx_sin[11..0], respectively. The digitized analog signal is represented by the ADC_FF[11..0] signalogram, and the results of its multiplication by the reference quadrature signals are represented by the multsin[23..0] and multcos[23..0] signalograms, respectively. The results of digital filtering, as a consequence of signal transformations in decimation and compensating filters, are shown on the signalograms CIC1[7..0], CIC2[7..0] and FIR1[7..0], FIR2[7..0]. Other signalograms in Figure 8 (clcbus[0], 15M, CLK, VCA_RX, VCA_TX, VCA[3..0]) visualize the auxiliary clock and the synchronization signals.

The results of previous studies using the SignalTap Logic Analyzer, conducted for the frequency range from 1 MHz to 50 MHz, confirmed the possibility of implementing the proposed methods for configuring an integrated computational core of a pulsed NQR sensor based on an FPGA.

**Figure 9:**
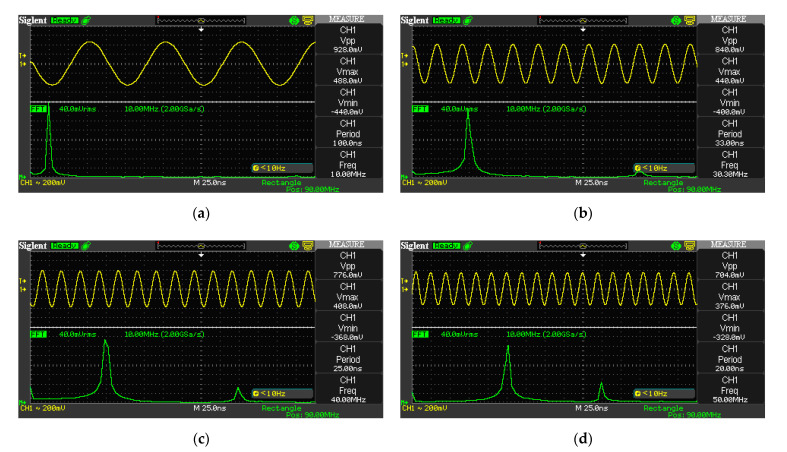
Experimental waveforms of signals generated by the first DDS channel of the proposed FPGA configuration structure: (**a**) 10 MHz; (**b**) 30 MHz; (**c**) 40 MHz; (**d**) 50 MHz.

The shape of the generated signals and their frequency characteristics were monitored in the frequency range of 1 MHz—50 MHz using a digital oscilloscope SIGLENT SDS 1202CNL+ with a bandwidth of 200 MHz and a sampling frequency of 2 GSa/s (Figure 9).

## 5. Conclusions

Using simulation tools, a method for the implementation of the configuration structure of an integrated computational core of a pulsed NQR sensor, implemented using programmable logic integrated circuits, was developed and experimentally investigated, and provided an improvement in a number of characteristics: a decrease in weight, size and cost indicators and an increase in configuration flexibility when changing the experimental conditions.

The configuration structures of functional modules were experimentally obtained, which made it possible to implement on an FPGA:

1. The synthesis of excitation pulses coherent with a carrier frequency in the range of 1 MHz–50 MHz with arbitrary time intervals due to the formation of sequences of codes of the instantaneous linear-variable phase of the signal by a three-channel 48 bit DDS, which differ from the known ones by minimizing the signal delay time to 20 ns in the structure of the programmable crystal.
2. The formation of 90° video pulses with a duration of 0.1 μs–100 μs; 180° video pulses with a duration of 0.2 μs–200 μs; the most relevant NQR excitation pulse sequences (including user ones with the number of pulses up to 32) and the setting of interpulse pauses with a duration of 0.1 μs–500 ms with an accuracy of 100 ns.
3. The digital quadrature reception of the FID signals in the frequency range of 1 MHz–50 MHz without the use of analog mixers and bandpass filters, which allows one to change the parameters of reconfigured decimation and compensating filters to increase the signal-to-noise ratio when registering NQR at low concentrations of the test samples.

## Figures and Tables

**Table 1:** Hardware connection mapping table.

Hardware	Functionality	FPGA in	FPGA out
DAC AD9772AASTZ	DDS control		DAC[13..0]
			DAC_CLKn
			DAC_CLKp
			DAC_RESET
LED			DDS_Act_LED
ADC AD9230BCPZ	DDC control	ADC[11..0]	ADC_CLKn
		DCOp	ADC_CLKp
		ORp	
		ORn	
LED			p_overload
LED			n_overload
DCA AD8369ARU	DCA control		DCA[3..0]
			DCA_RX
			DCA_TX
USB FIFO FT2232HL	DAQ control	USB_SYNC	ADD[7..0]
RF transmitter	Tx synchronization		TTL
RF preamplifier	Rx synchronization		TTL_Receiver
Control unit	User interface	In_DATA_BUS[3..0]	
		In_DATA_CLK	
		In_DATA_SYNC	
		CLK	
